# The complete chloroplast genome and phylogenetic analysis of *Cyananthus macrocalyx* Franch. 1887 Campanulaceae

**DOI:** 10.1080/23802359.2025.2503403

**Published:** 2025-05-11

**Authors:** Hai-Tao Ma, Dan Lei, Qi-Yin Chen, Bei Jiang, Yong-Zeng Zhang

**Affiliations:** a Yunnan Key Laboratory of Screening and Research on Anti-pathogenic Plant Resources from Western Yunnan, Dali, China; b College of Pharmacy & Institute of Materia Medica, Dali University, Dali, China

**Keywords:** *Cyananthus macrocalyx*, chloroplast complete genome, *Cyananthus*, phylogenetic analysis

## Abstract

*Cyananthus macrocalyx* Franch. belongs to the genus *Cyananthus* in the family Campanulaceae. In this study, we sequenced and analyzed its chloroplast genome then constructed a phylogenetic tree to determine its phylogenetic position. The results show: the total length of the chloroplast genome of *C. macrocalyx* was 167,964 bp, with a GC content of 37.95%, and the chloroplast genome exhibited a standard quadripartite structure, consisting of a large single-copy (LSC) region (82,884 bp), a small single-copy (SSC) region (8118 bp), and a pair of inverted repeats (IRs) regions (76,962 bp). A total of 112 genes were annotated from *C. macrocalyx*, including 78 protein-coding genes, 30 transfer RNA (tRNA) genes, and four ribosomal RNA (rRNA) genes. Additionally, 54 simple sequence repeats (SSRs) were detected, most of which were A/T monomeric sequences. Phylogenetic analyses showed that *C. macrocalyx* is clustered with *C. flavus*, suggesting a close relationship between the two species. This study provides a valuable resource for the genetics and evolution study of the *Cyananthus*.

## 1. Introduction

The genus *Cyananthus* of the family Campanulaceae is classified as an annual and perennial herb (Hong and Ma 1991). It comprises about 18 species worldwide and 17 species in China (Hong et al. 2011), is an endemic genus of the Himalayan and the Hengduan Mountains (Zhou et al. 2013). The genus *Cyananthus* has significant pharmacological value. For example, *C. incanus* and *C. hookeri* are used to treat damp-heat (Lin and Huang 1989). The whole herb of *C. inflatus* was used as a medicine to treat infantile convulsion and rheumatic disorder (Lin and Huang 1989).

*Cyananthus* originated in the Kan-Dian ancient landmass (which is presently in Western Sichuan and Northwest Yunnan, covering almost the entire range of the Hengduan Mountains) in the early Tertiary (Hong and Ma 1991), and was adapted to the Himalayan region following the uplift of the Tibetan Plateau in the middle and late Tertiary (Zhou et al. 2013). *Cyananthus* was one of the most primitive genera in the Campanulaceae, for its special flower character (Hong and Ma 1991; Hong 1995). However, only two species (*C. flavus* and *C. lobatus*) of complete chloroplast genome data have been deposited in NCBI so far and the chloroplast genome of *C. macrocalyx* has not been reported. In this study, the chloroplast genome of *C. macrocalyx* was successfully assembled and analyzed, aiming to provide evidence for further studies on *Cyananthus* in species identification, phylogeny, and species conservation.

## 2. Materials and methods

### 2.1. Sampling

**Figure 1.**
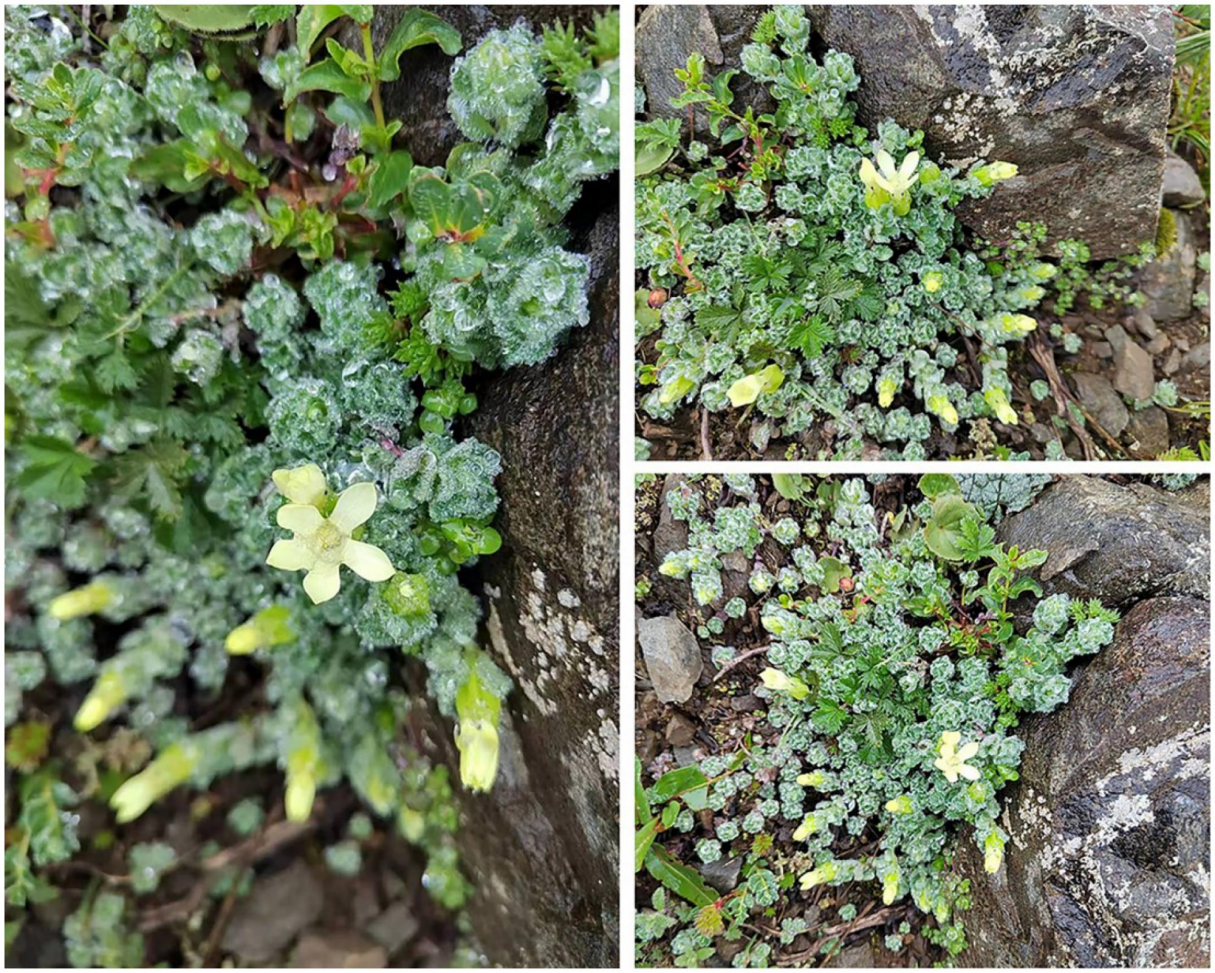
*Cyananthus macrocalyx*. The photo was taken by Yong-Zeng Zhang in Baima Mountain, Yunnan, China. The flowers are yellow-green and solitary at the stem apex, with leaves usually opposite or alternate, and four or five leaves clustered in a whorled arrangement beneath the flower.

Fresh leaves of *Cyananthus macrocalyx* were collected from Baima Mountain (Deqin, Yunnan, China; coordinates: 28°20′10.896″N, 99°4′58.332″E) by Dr. Yong-Zeng Zhang (Figure 1), and subsequently desiccated using silica gel. The voucher specimen has been archived in the Key Laboratory of Screening and Research on Anti-pathogenic Plant Resources from Western Yunnan (voucher code 20230724-4, Yong-Zeng Zhang, cell_zyz@163.com).

### 2.2. DNA extraction and sequencing

Chloroplast genomic DNA was isolated with a modified CTAB method (Doyle 1987). DNA quality and concentration were assessed by 1% agarose gel electrophoresis and spectrophotometry (Bio-Rad, Hercules, CA). The DNA was sheared to obtain a fragment of approximately 350 bp for library construction. Sequencing of DNA libraries on the DNBSEQ-T7 sequencing platform. The fastp v0.23.2 (Chen 2023) was then used to remove low-quality sequences. The sequencing depth coverage was conducted by Samtools (Li et al. 2009). The entire sequencing process was carried out by Benagen Technology (Wuhan, China).

### 2.3. Assembly and annotation

We performed a *de novo* assembly of the complete chloroplast genome of *Cyananthus macrocalyx*. The complete chloroplast genome was assembled using GetOrganelle v1.7.5 (Jin et al. 2020). The chloroplast complete genome was annotated using CPGAVAS2 (Shi et al. 2019), and the chloroplast genome map was visualized using OGDRAW (Greiner et al. 2019). Each annotation error of the chloroplast genome was manually modified and corrected using CPGView (Liu et al. 2023) and Apollo (Lewis et al. 2002). Finally, we submitted the annotated genomic sequence to GenBank with the accession number PQ584750.1.

### 2.4. Repeat sequence analysis

The repeated sequence of *Cyananthus macrocalyx* was analyzed using microsatellite (Beier et al. 2017), tandem repeats finder (Benson 1999), and REPuter (Kurtz et al. 2001), respectively.

### 2.5. Phylogenetic analysis

To investigate the phylogenetic relationships of *Cyananthus macrocalyx*, the chloroplast complete genomes of 25 species of the family Campanulaceae were downloaded from the NCBI database, and *Wahlenbergia marginata*, *Campanula pallida*, and *Asyneuma japonicum* were selected as outgroups for analysis. Phylogenetic analyses were performed using maximum-likelihood (ML) and Bayesian inference (BI) methods. All the sequences were aligned using MAFFT v7.313 (Katoh and Standley 2013) and automatic alignment trimming sequences with Trimal v1.4 (Capella-Gutiérrez et al. 2009). Based on ModelFinder (Kalyaanamoorthy et al. 2017) in PhyloSuite v1.2.3 (Zhang et al. 2020), the best model for ML was filtered: GTR + I + G4 and BI best model: GTR + F + I + G. IQ-tree 2.2.0 (Nguyen et al. 2015) was used to construct a phylogenetic tree with 1000 bootstraps based on the ML method (Minh et al. 2013). Bayesian inference phylogeny (two parallel runs, 1,000,000 generations) based on MrBayes v3.2.7 (Ronquist et al. 2012), where an initial 25% of the sampled data was discarded as burn-in data. The phylogenetic tree was visualized using Figtree v1.4.4 (Rambaut 2018).

## 3. Results

**Figure 2.**
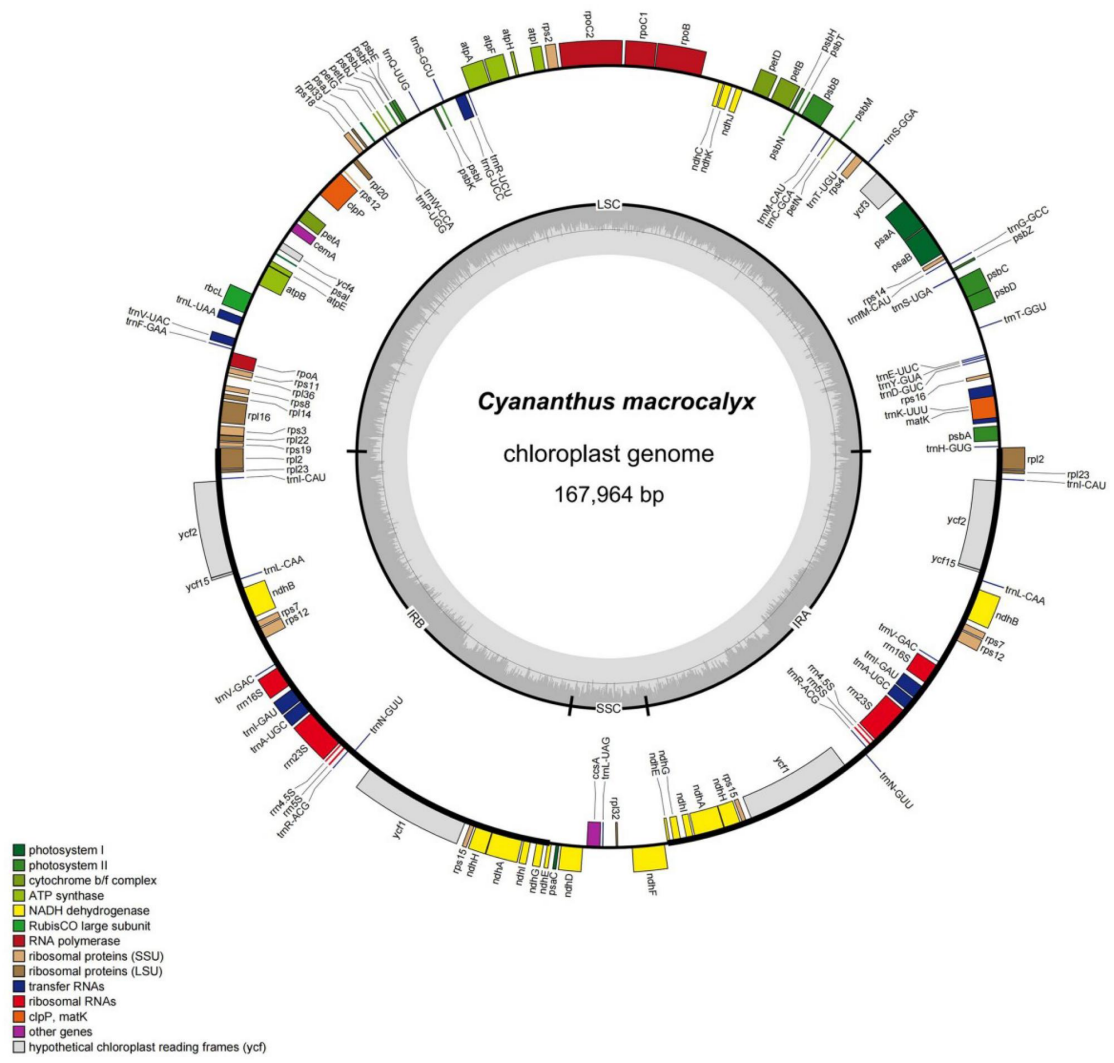
Circular map of the *Cyananthus macrocalyx* chloroplast genome. Genes shown inside the circle are transcribed clockwise, those outside the circle are counterclockwise transcribed. The light grey and the darker grey in the inner circle represent AT and GC content, respectively. Different functional groups of genes are signed according to the colored boxes. LSC: large single copy; SSC: small single copy; IRA/IRB: inverted repeat regions.

The chloroplast complete genome of *Cyananthus macrocalyx* exhibited a typical quadripartite structure, which was 167,964 bp in length with 37.95% GC content (Figure 2). The minimum and average read mapping depths were 465× and 9735.42× (Figure S1). The chloroplast complete genome consisted of a large single-copy region (LSC) of 82,884 bp, a small single-copy (SSC) region of 8118 bp, and a pair of inverted repeat regions (IRA and IRB) of 76,962 bp (Figure 2). A total of 112 genes were annotated, including 78 protein-coding, 30 transfer RNA (tRNA), and four rRNA genes. In the genome, seven protein-coding genes (*ndhA*, *ndhB*, *rpl2*, *rpl16*, *petB*, *petD*, and *atpF*) and six tRNA genes (*trn*A-UGC, *trnG-UCC*, *trnI-GAU*, *trn*K-UUU, *trn*L-UAA, and *trn*V-UAC) contain an intron. In addition, three genes (*ycf3*, *clpP*, and *rps12*) contain two introns (Table S1). Notably, the chloroplast genome contained nine cis-splicing genes (Figure S2) and one trans-splicing gene (Figure S3). In addition, 54 simple sequence repeats (SSRs) were detected, containing monomeric (22), dimeric (12), trimeric (7), tetrameric (8), pentameric (2), and hexameric (3) and long sequence repeats (LSRs) containing 641 forward repeats, 618 palindromic repeats, 12 reverse repeats, and 13 complement repeats (Figure S4).

**Figure 3.**
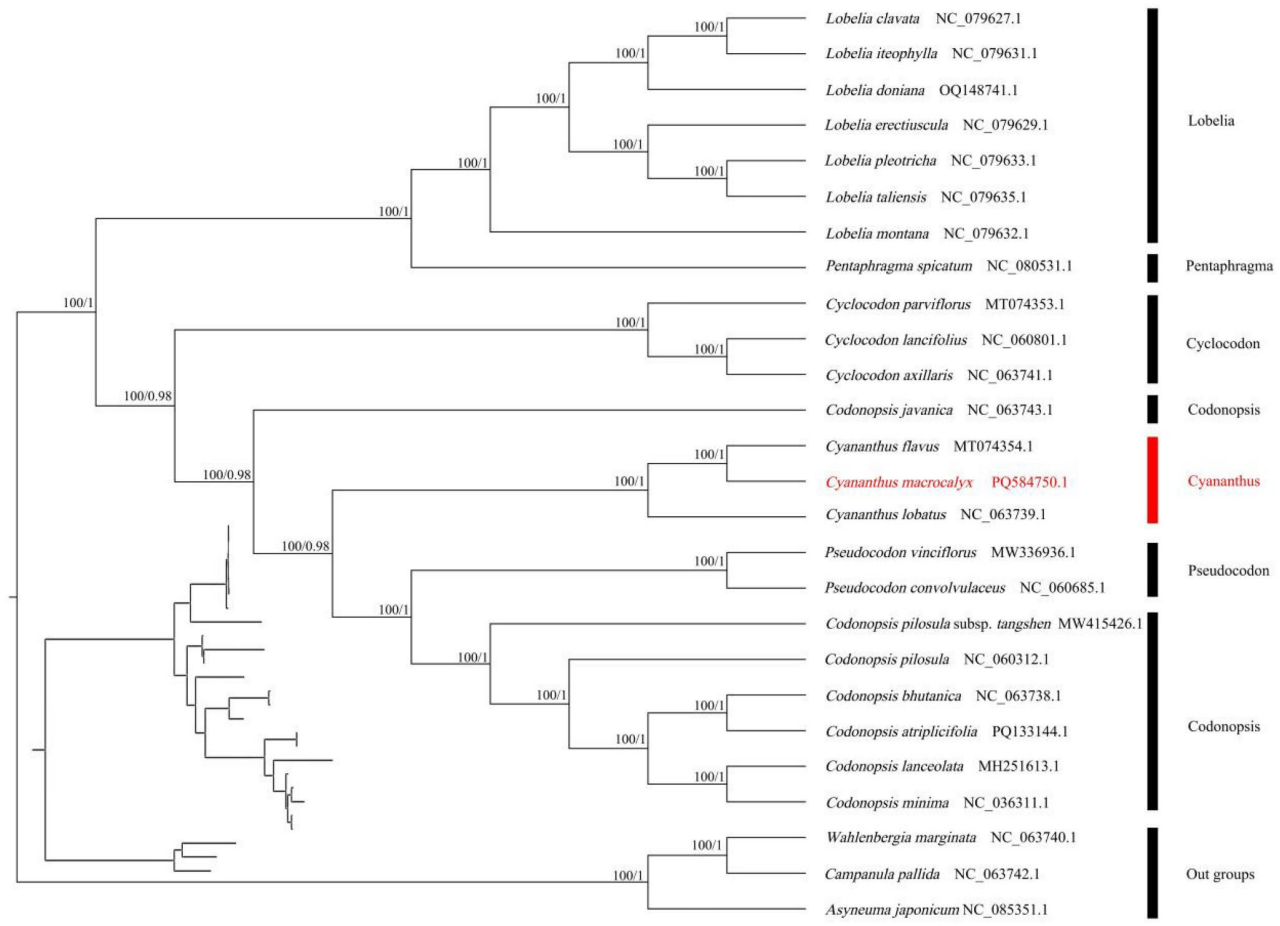
Maximum-likelihood (ML) and Bayesian inference (BI) phylogenetic tree based on the complete chloroplast genome sequence of 26 species from the Campanulaceae. Number above nodes are support values with ML bootstrap (BS) values on the left and BI posterior probabilities (PPs) values on the right. The sequences used for constructing the phylogenetic tree are as follows: *Cyananthus macrocalyx* PQ584750.1, *Cyananthus flavus* MT074354.1 (Li et al. 2020), *Cyananthus lobatus* NC_063739.1, *Cyclocodon parviflorus* MT074353.1 (Li et al. 2020), *Cyclocodon lancifolius* NC_060801.1 (Shang et al. 2020), *Cyclocodon axillaris* NC_063741.1, *Codonopsis bhutanica* NC_063738.1, *Codonopsis javanica* NC_063743.1, *Codonopsis atriplicifolia* PQ133144.1, *Codonopsis pilosula* subsp. *tangshen* MW415426.1, *Pseudocodon convolvulaceus* NC_060685.1, *Codonopsis minima* NC_036311.1 (Cheon et al. 2017), *Codonopsis pilosula* NC_060312.1, *Codonopsis lanceolata* MH251613.1, *Lobelia clavata* NC_079627.1, *Lobelia iteophylla* NC_079631.1 (Li et al. 2023), *Lobelia doniana* OQ148741.1 (Li et al. 2023), *Lobelia montana* NC_079632.1 (Li et al. 2023), *Lobelia taliensis* NC_079635.1 (Li et al. 2023), *Lobelia erectiuscula* NC_079629.1 (Li et al. 2023), *Lobelia pleotricha* NC_079633.1 (Li et al. 2023), *Pentaphragma spicatum* NC_080531.1 (Cheng et al. 2023), *Pseudocodon vinciflorus* MW336936.1, *Wahlenbergia marginata* NC_063740.1, *Campanula pallida* NC_063742.1, and *Asyneuma japonicum* NC_085351.1. The chloroplast genomes of *Cyananthus macrocalyx* in this study were labeled in red color.

ML and BI trees indicate the phylogenetic relationships of *Cyananthus macrocalyx* within the Campanulaceae family. The phylogenetic analysis showed that the species of Campanulaceae and three outgroup species each form a highly supported monophyletic group (Figure 3). Three species of *Cyananthus* formed a strongly monophyletic clade and *C. macrocalyx* and *C. flavus* were sister species to each other (Figure 3).

## 4. Discussion and conclusions

In this study, the complete chloroplast genomes of *C. macrocalyx* were reported for the first time and contained 112 unique genes, including 78 protein-coding genes, 30 tRNA genes, and four rRNA genes. The chloroplast genome of *C. macrocalyx* is similar in character to that of *C. flavus* and *C. lobatus*, suggesting that *Cyananthus* chloroplast genomes are relatively evolutionarily conserved. Moreover, a total of 54 SSRs were detected in the chloroplast genome of the *C. macrocalyx*. Among them, the mononucleotide was the most abundant, and dominated by adenine (A) and thymine (T). This indicates the preference of A and T bases for SSR in the *Cyananthus*.

The phylogenetic analysis showed that three species of *Cyananthus* are clustered into a single group, and *C. macrocalyx* is more closely related to *C. flavus*, which is similar to the work of Zhou et al. (2013). In addition, our results of the relationship of *Pseudocodon* and *Codonopsis*, and classification of *P. vinciflorus* and *P. convolvulaceus* were similar to Wang and Hong (2015). It is worth noting that *Codonopsis javanica* was not clustered in *Codonopsis* (Figure 3), indicating that *Codonopsis* needs to be further studied. All in all, our study enriches the molecular biological resources of *Cyananthus* and provides evidence for further species identification, resource conservation and systematic evolution of *Cyananthus*.

## Supplementary Material

supplementary material clean copy.docx

## Data Availability

The genome sequence data that support the findings of this study are openly available in GenBank (NCBI, https://www.ncbi.nlm.nih.gov/) under the accession number PQ584750.1. The associated BioProject, SRA, and Bio-Sample numbers are PRJNA1185496, SRR31359883, and SAMN44706201, respectively.

## References

[CIT0001] Beier S, Thiel T, Münch T, Scholz U, Mascher M. 2017. MISA-web: a web server for microsatellite prediction. Bioinformatics. 33(16):2583–2585. doi:10.1093/bioinformatics/btx198. 28398459 PMC5870701

[CIT0002] Benson G. 1999. Tandem repeats finder: a program to analyze DNA sequences. Nucleic Acids Res. 27(2):573–580. doi:10.1093/nar/27.2.573. 9862982 PMC148217

[CIT0003] Capella-Gutiérrez S, Silla-Martínez JM, Gabaldón T. 2009. trimAl: a tool for automated alignment trimming in large-scale phylogenetic analyses. Bioinformatics. 25(15):1972–1973. doi:10.1093/bioinformatics/btp348. 19505945 PMC2712344

[CIT0004] Chen S. 2023. Ultrafast one-pass FASTQ data preprocessing, quality control, and deduplication using fastp. Imeta. 2(2):e107. doi:10.1002/imt2.107. 38868435 PMC10989850

[CIT0005] Cheng Z, Yu B, Huang X, Bin Z, Liu SZ, Chi Z. 2023. The first complete chloroplast genome sequence of *Pentaphragma spicatum* Merr. (Pentaphragmataceae) and phylogenetic analysis. Mitochondrial DNA B Resour. 8(12):1368–1372. doi:10.1080/23802359.2023.2290339. 38196793 PMC10776070

[CIT0006] Cheon KS, Kim KA, Han JS, Yoo KO. 2017. The complete chloroplast genome sequence of *Codonopsis minima* (Campanulaceae), an endemic to Korea. Conserv Genet Resour. 9(4):541–543. doi:10.1007/s12686-017-0718-0.

[CIT0007] Doyle J. 1987. A rapid DNA isolation procedure for small quantities of fresh leaf tissue. Phytochem Bull. 19(1):11–15.

[CIT0008] Greiner S, Lehwark P, Bock R. 2019. OrganellarGenomeDRAW (OGDRAW) version 1.3.1: expanded toolkit for the graphical visualization of organellar genomes. Nucleic Acids Res. 47(W1):W59–W64. doi:10.1093/nar/gkz238. 30949694 PMC6602502

[CIT0009] Hong DY, Ge S, Lammers TG, Klein LL. 2011. Campanulaceae. In: Wu ZY, Hong DY, Raven, PH, editors. Flora of China. Vol. 19. St. Louis and Beijing: Missouri Botanical Garden Press and Science Press; p. 505–512.

[CIT0010] Hong DY, Ma LM. 1991. Systematics of the genus *Cyananthus* Wall. Acta Phytotax. 29(1):25–51.

[CIT0011] Hong DY. 1995. The geography of the Campanulaceae: on the distribution centres. Syst Evol. 33(6):521–536.

[CIT0012] Jin JJ, Yu WB, Yang JB, Song Y, dePamphilis CW, Yi TS, Li DZ. 2020. GetOrganelle: a fast and versatile toolkit for accurate de novo assembly of organelle genomes. Genome Biol. 21(1):241. doi:10.1186/s13059-020-02154-5. 32912315 PMC7488116

[CIT0013] Kalyaanamoorthy S, Minh BQ, Wong TKF, Von Haeseler A, Jermiin LS. 2017. ModelFinder: fast model selection for accurate phylogenetic estimates. Nat Methods. 14(6):587–589. doi:10.1038/nmeth.4285. 28481363 PMC5453245

[CIT0014] Katoh K, Standley DM. 2013. MAFFT multiple sequence alignment software version 7: improvements in performance and usability. Mol Biol Evol. 30(4):772–780. doi:10.1093/molbev/mst010. 23329690 PMC3603318

[CIT0015] Kurtz S, Choudhuri JV, Ohlebusch E, Schleiermacher C, Stoye J, Giegerich R. 2001. REPuter: the manifold applications of repeat analysis on a genomic scale. Nucleic Acids Res. 29(22):4633–4642. doi:10.1093/nar/29.22.4633. 11713313 PMC92531

[CIT0016] Lewis SE, Searle SMJ, Harris N, Gibson M, Lyer V, Richter J, Wiel C, Bayraktaroglu L, Birney E, Crosby MA, et al. 2002. Apollo: a sequence annotation editor. Genome Biol. 3(12):RESEARCH0082. doi:10.1186/gb-2002-3-12-research0082. 12537571 PMC151184

[CIT0017] Li CJ, Wang RN, Li DZ. 2020. Comparative analysis of plastid genomes within the Campanulaceae and phylogenetic implications. PLOS One. 15(5):e0233167. doi:10.1371/journal.pone.0233167. 32407424 PMC7224561

[CIT0018] Li CJ, Xie XT, Liu HX, Wang RN, Li DZ. 2023. Plastome evolution in the East Asian lobelias (Lobelioideae) using phylogenomic and comparative analyses. Front Plant Sci. 14:1144406. doi:10.3389/fpls.2023.1144406. 37063184 PMC10102522

[CIT0019] Li H, Handsaker B, Wysoker A, Fennell T, Ruan J, Homer N, Marth G, Abecasis G, Durbin R, 1000 Genome Project Data Processing Subgroup. 2009. The Sequence Alignment/Map format and SAMtools. Bioinformatics. 25(16):2078–2079. doi:10.1093/bioinformatics/btp352. 19505943 PMC2723002

[CIT0020] Lin QW, Huang WL. 1989. Campanulaceae. In: Li YK, editor. Flora of Guizhou. Vol. 6. Sichuan: Sichuan Nationalities Publishing House; p. 362–410.

[CIT0021] Liu SY, Ni Y, Li JL, Zhang XY, Yang HY, Chen HM, Liu C. 2023. CPGView: a package for visualizing detailed chloroplast genome structures. Mol Ecol Resour. 23(3):694–704. doi:10.1111/1755-0998.13729. 36587992

[CIT0022] Minh BQ, Nguyen MAT, Von Haeseler A. 2013. Ultrafast approximation for phylogenetic bootstrap. Mol Biol Evol. 30(5):1188–1195. doi:10.1093/molbev/mst024. 23418397 PMC3670741

[CIT0023] Nguyen LT, Schmidt HA, Haeseler AV, Minh BQ. 2015. IQ-TREE: a fast and effective stochastic algorithm for estimating maximum-likelihood phylogenies. Mol Biol Evol. 32(1):268–274. doi:10.1093/molbev/msu300. 25371430 PMC4271533

[CIT0024] Rambaut A. 2018. FigTree v 1.4.4. Edinburgh: University of Edinburgh.

[CIT0025] Ronquist F, Teslenko M, Van Der Mark P, Ayres DL, Darling A, Höhna S, Larget B, Liu L, Suchard MA, Huelsenbeck JP. 2012. MrBayes 3.2: efficient Bayesian phylogenetic inference and model choice across a large model space. Syst Biol. 61(3):539–542. doi:10.1093/sysbio/sys029. 22357727 PMC3329765

[CIT0026] Shang FN, Fang HL, Qian J, Duan BZ. 2020. High-throughput sequencing of complete chloroplast genome of Yi folk medicine *Cyclocodon lancifolius* (Roxb.) Kurz. Mitochondrial DNA B. 5(2):1791–1793. doi:10.1080/23802359.2020.1749178.

[CIT0027] Shi LC, Chen HM, Jiang M, Wang LQ, Wu X, Huang LF, Liu C. 2019. CPGAVAS2, an integrated plastome sequence annotator and analyzer. Nucleic Acids Res. 47(W1):W65–W73. doi:10.1093/nar/gkz345. 31066451 PMC6602467

[CIT0028] Wang Q, Hong DY. 2015. Taxonomic revision of the genus Pseudocodon (Campanulaceae) based on character analysis and molecular phylogeny. Phytotaxa. 204(1):49–64. doi:10.11646/phytotaxa.204.1.4.

[CIT0029] Zhang D, Gao F, Jakovlić I, Zou H, Zhang J, Li WX, Wang GT. 2020. PhyloSuite: an integrated and scalable desktop platform for streamlined molecular sequence data management and evolutionary phylogenetics studies. Mol Ecol Resour. 20(1):348–355. doi:10.1111/1755-0998.13096. 31599058

[CIT0030] Zhou Z, Hong DY, Niu Y, Li GD, Nie ZL, Wen J, Sun H. 2013. Phylogenetic and biogeographic analyses of the Sino-Himalayan endemic genus *Cyananthus* (Campanulaceae) and implications for the evolution of its sexual system. Mol Phylogenet Evol. 68(3):482–497. doi:10.1016/j.ympev.2013.04.027. 23669010

