# Supplementary material for: The complete chloroplast genome and phylogenetic analysis of Cyananthus macrocalyx Franch. 1887 Campanulaceae
Source: Mitochondrial DNA B Resour. 2025 May 11;10(6):442–6. doi: 10.1080/23802359.2025.2503403 (PMC12077473; doi:10.1080/23802359.2025.2503403)
Supplement: supplementary material clean copy.docx [file TMDN_A_2503403_SM5450.docx]

**Figure S1.** The Map of Sequencing Depth and Coverage, representing the sequencing depth across the chloroplast genome. The x-axis shows the genomic position (in base pairs, bp), and the y-axis shows the sequencing depth (X). The blue line indicates the sequencing depth at each genomic position


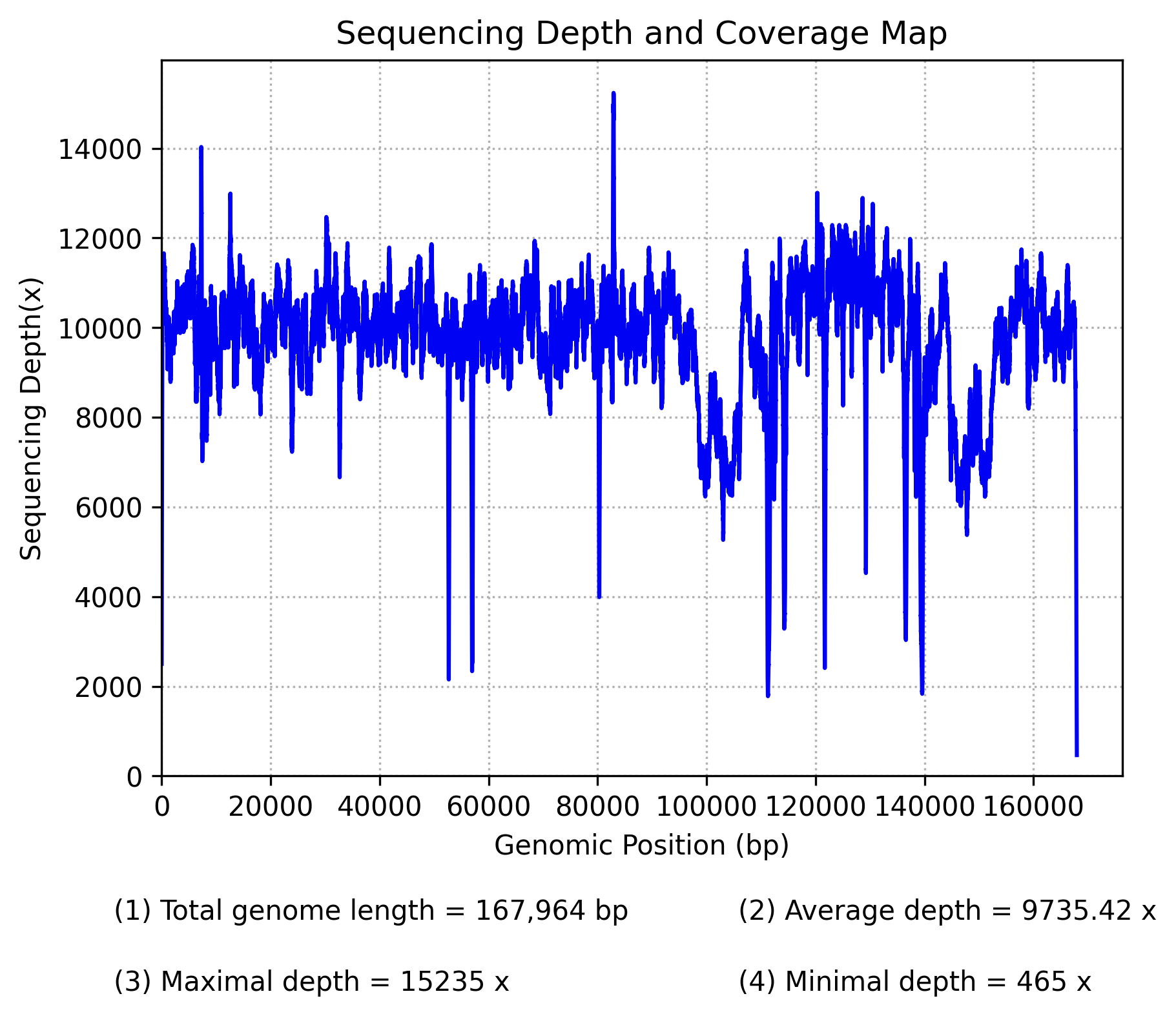


**Figure S2.** The map of the cis-splicing genes, including nine cis-splicing genes (*ycf3, petB, petD, atpF, clpP, rpl16, rpl2, ndhB, ndhA*), the *ndhB* and *ndhA* were duplicates, seven of them have one intron and two exons, and two have two introns and three exons.


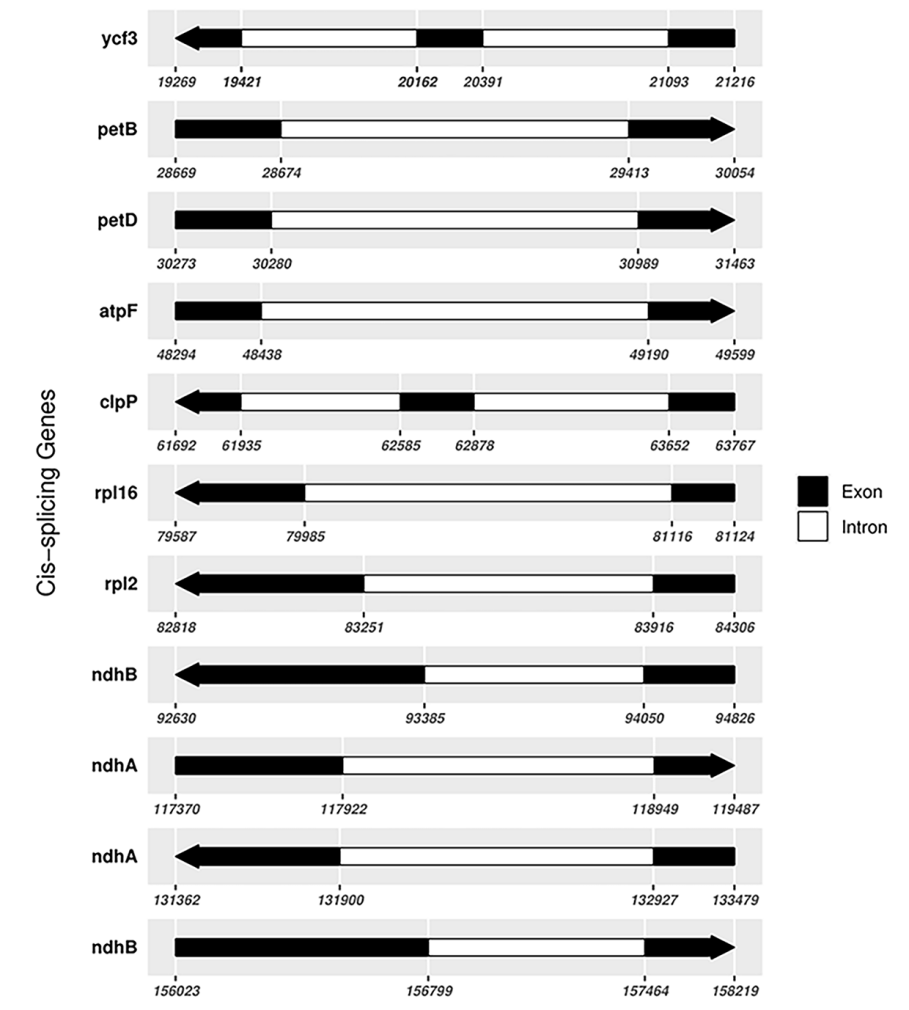


**Figure S3.** The map of the trans-splicing gene *rps12* in the *Cyananthus macrocalyx* chloroplast genome.


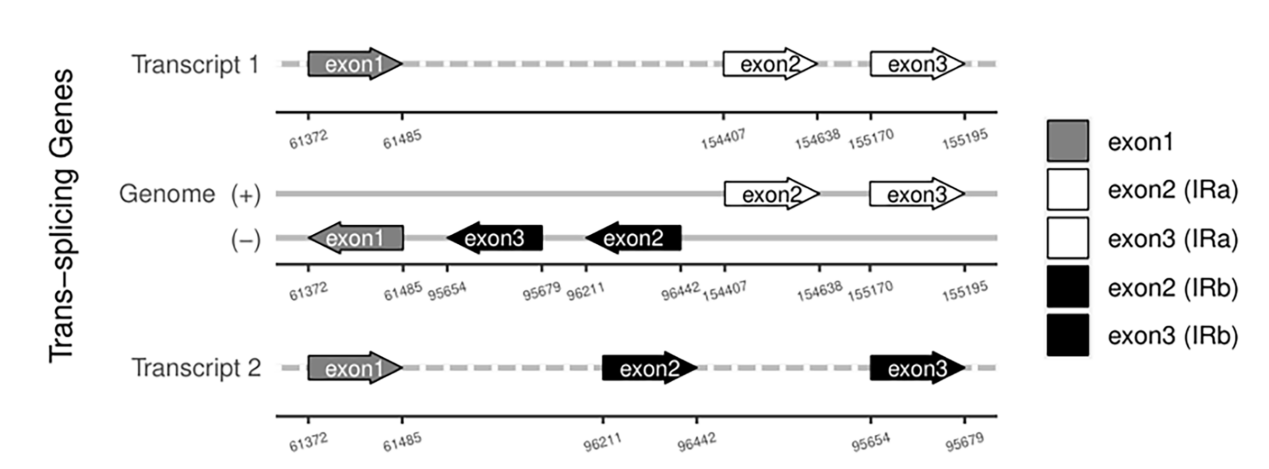


**Figure S4.** The map of the Repeat sequence analysis. For A map, x-axis shows the type of SSRs, green representative mononucleotide, blue representative dinucleotide, red representative trinucleotide, yellow representative tetranucleotide, purple representative pentanucleotide, grey representative hexanucleotide and the y-axis shows the number of repeats. For B map, x-axis shows the type of repeats, yellow representative tandem repeats, blue representative Palindromic repeats, orange representative forward repeats, purple representative reverse repeats, red representative Complementary repeats and the y-axis shows the number of repeats.


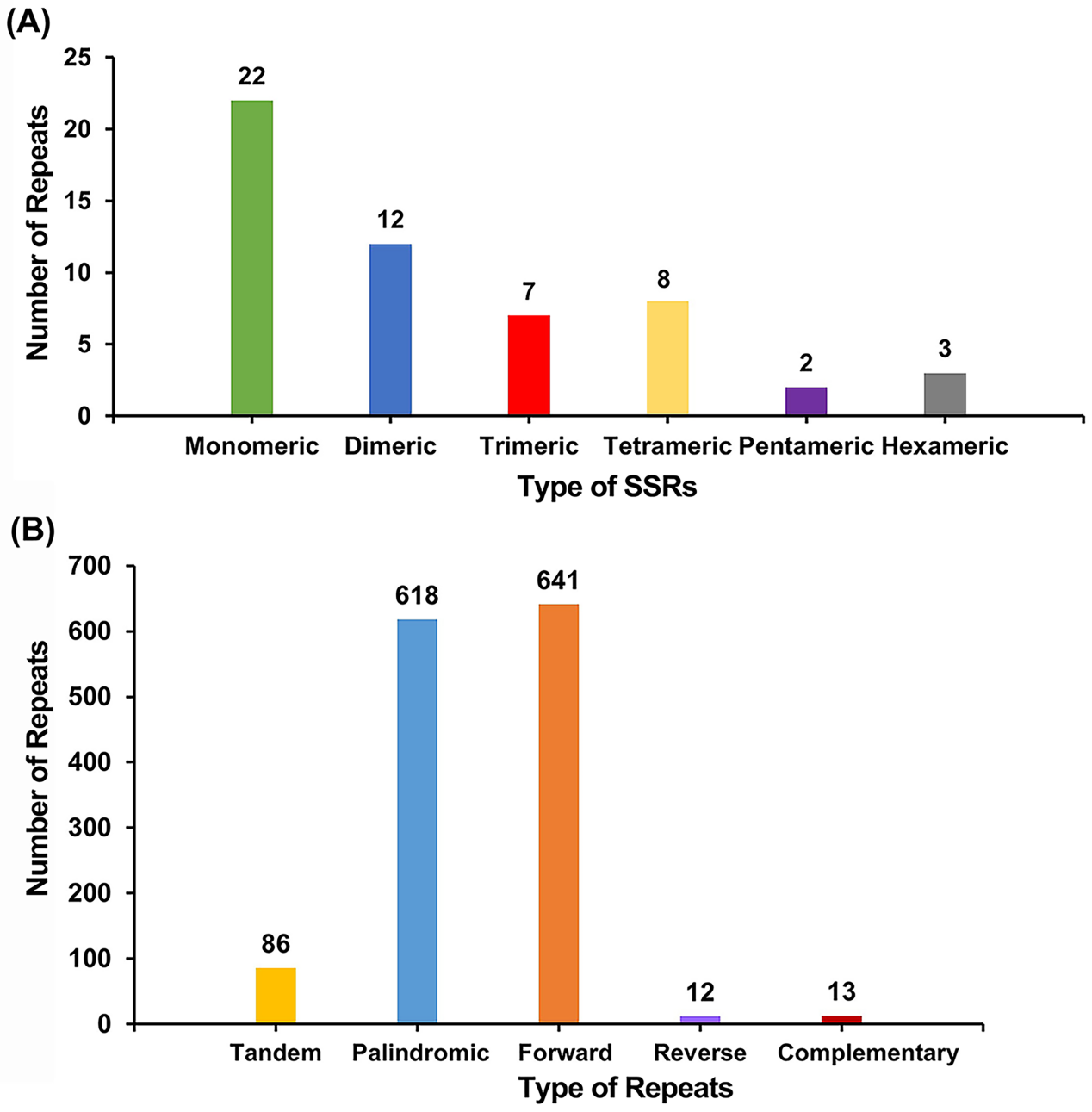


**Table S1.** The chloroplast-encoded genes of *Cyananthus macrocalyx*

Note: The number in brackets is the number of copies of the gene, (×2) means there are two copies. Asterisks indicate intron-containing genes.

| Category for gene | Group of genes | Name of genes |
| --- | --- | --- |
| Gene for  photosynthesis | Subunits of NADH-dehydrogenase | *ndhA*(×2)*， *ndhB*(×2)*， *ndhC*， *ndhD*， *ndhE*(×2)， *ndhF*， *ndhG*(×2)， *ndhH*(×2)， *ndhI*(×2)， *ndhJ*， *ndhK* |
|  | Subunits of photosystem Ⅰ | *psaA*, *psaB*, *psaC*, *psaI*, *psaJ*, *psbA*, *psbB*, *psbC*, *psbD*, *psbE*, *psbF*, *psbH* |
|  | Subunits of photosystem Ⅱ | *psb*I, *psb*J, *psb*K, *psb*L, *psb*M, *psb*N, *psb*T, *psb*Z, *ycf*3** |
|  | Subunits of cytochrome b/f complex | *petA*, *petB**, *petD**, *petG*, *petL*, *petN* |
|  | Subunits of ATP synthase | *atpA*, *atpB*, *atpE*, *atpF**, *atpH*, *atpI* |
|  | Large subunit of rubisco | *rbcL* |
| Self-replication | Small subunit of ribosome | *rps2*, *rps3*, *rps4*, *rps7*(×2), *rps8*, *rps11*,  *rps12*(×2)**, *rps14*, *rps15*(×2), *rps16*, *rps18*,  *rps19* |
|  | Large subunit of ribosome | *rpl2*(×2)*, *rpl14*, *rpl16**, *rpl20*, *rpl22*, *rpl23*(×2),  *rpl32*, *rpl33*, *rpl36* |
|  | DNA dependent RNA polymerase | *rpoA*, *rpoB*, *rpoC1*, *rpoC2* |
|  | tRNA genes | *trnA-UGC*(×2)*, *trnC-GCA*, *trnD-GUC*, *trnE-UUC*, *trnF-GAA*, *trnfM-CAU*, *trnG-GCC*, *trnG-UCC**, *trnH-GUG*, *trnI-CAU*(×2), *trnI-GAU*(×2)*, *trnK-UUU**, *trnL-CAA*(×2), *trnL-UAA**, *trnL-UAG*, *trnM-CAU*, *trnN-GUU*(×2), *trnP-UGG*, *trnQ-UUG*, *trnR-ACG*(×2), *trnR-UCU*, *trnS-GCU*, *trnS-GGA*, *trnS-UGA*, *trnT-GGU*, *trnT-UGU*, *trnV-GAC*(×2), *trnV-UAC**, *trnW-CCA*, *trnY-GUA* |
|  | rRNA genes | *rrn4.5S*(×2), *rrn5S*(×2), *rrn16S*(×2), *rrn23S*(×2) |
| Other genes | Maturase | *matK* |
|  | c-type cytochrom synthesis gene | *ccsA* |
|  | Envelope membrane protein | *cemA* |
|  | Protease | *clpP*** |
|  | Genes of unknown functions Open Reading | *ycf1*(×2), *ycf2*(×2), *ycf4*, *ycf15*(×2) |
